# Supplementary material for: Fine-tuning protein language models unlocks the potential of underrepresented viral proteomes
Source: PeerJ. 2025 Sep 18;13:e19919. doi: 10.7717/peerj.19919 (PMC12450373; doi:10.7717/peerj.19919)
Supplement: Supplemental Information 1 [file peerj-13-19919-s001.pdf]

# Fine-Tuning Protein Language Models Unlocks the Potential of Underrepresented Viral Proteomes

**Rajan Sawhney**[1], **Barbra D. Ferrell**[2], **Thibaut Dejean**[1], **Zachary Schreiber**[2, 5], **William Harrigan**[3], **Shawn W. Polson**[2, 5], **K. Eric Wommack**[4, 5], **Mahdi Belcaid**[1]

[1]Department of Information and Computer Sciences, University of Hawai'i at Manoa
[2]Department of Computer and Information Sciences, University of Delaware
[3]Hawai'i Institute of Marine Biology, University of Hawai'i at Manoa
[4]Department of Plant and Soil Sciences, University of Delaware
[5]Delaware Biotechnology Institute, University of Delaware

## SUPPLEMENTARY MATERIAL

### Experiment 1: Pairwise comparison-based experiments

#### *1.1 ECDF of pairwise cosine similarity distribution*

Provided below is the empirical cumulative distribution function (ECDF) plot of the cosine similarity distribution (Fig. S1). This accompanies the cosine similarity distribution box plot shown in Fig. 2.

#### *1.2 Average pairwise protein similarity using global sequence alignments with the BLOSUM62 scoring matrix*

We observed that pre-trained ESM2-3B yields inflated cosine similarity scores for pooled sequence embeddings when compared against the average pairwise protein similarity computed using global sequence alignments with the BLOSUM62 scoring matrix. For each protein Viral Orthologous Group (VOG), we aligned ten randomly selected sequences (same sequence used to compute pairwise sequence cosine similarities from the VOG test dataset) using the Biopython pairwise2 module. The alignment scores were normalized by the maximum sequence length for each pair to obtain a similarity value between 0 and 1. Mean similarity scores were computed per VOG and averaged across all groups to assess sequence redundancy and conservation within the dataset. This approach resulted in an average pairwise protein similarity score of 0.51, which is considerably low compared to the elevated cosine similarity score of 0.99 obtained via pre-trained ESM2-3B embeddings.

### Experiment 2: Clustering-based experiments

#### *2.1 DNA helicase domain analysis and phylogenetic tree*

**Pfam domain calculation**

Each of the 174 DNA helicase amino acid sequences were analyzed using InterProScan Tool version 5.67-99.0 with default parameters, querying against the Pfam database. The output file generated consists of all significant domain hits for each open reading frame (ORF) ID, along with the start and stop regions of where the domains are aligned to the reference sequence.

**Phylogentic tree**

The DNA helicase amino acid sequences were aligned using MAFFT –auto and their alignments were analyzed using FastTree (version 2.1) with the default parameters to generate an approximate maximum-likelihood tree newick file of their protein alignments. The newick file along with a file containing contextual domain metadata was then loaded into the Iroki tree visualization web tool to generate the final tree.

### Experiment 3: Alignment-based experiments

#### *3.1 Quality of vcMSA for bacteriophage proteins*

Mutual information and occupancy for vcMSA generated for UvsW helicase proteins from bacteriophages was derived using ProDy version 2.4.1 (Bakan et al., 2011; Zhang et al., 2021). Mutual information was

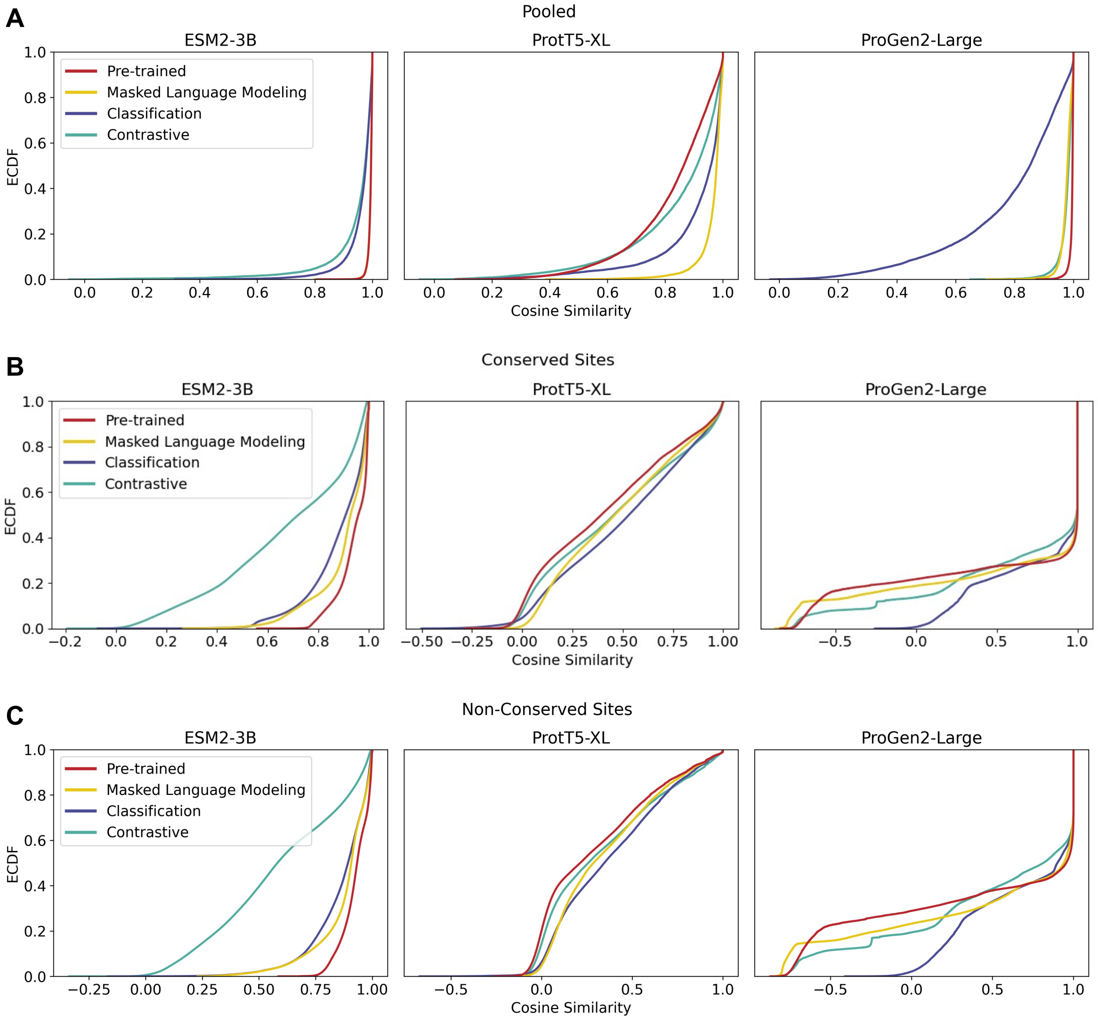


**Figure S1.** ECDF of pairwise cosine similarity values for (A) pooled sequence embeddings of sequences within an orthologous group, (B) conserved sites within multiple sequence alignments, and (C) non-conserved sites within multiple sequence alignments.

normalized using minimum entropy to account for sequence variability and to highlight relevant residue correlations.

## REFERENCES

Bakan, A., Meireles, L. M., and Bahar, I. (2011). Prody: protein dynamics inferred from theory and experiments. *Bioinformatics*, 27(11):1575–1577.

Zhang, S., Krieger, J. M., Zhang, Y., Kaya, C., Kaynak, B., Mikulska-Ruminska, K., Doruker, P., Li, H., and Bahar, I. (2021). Prody 2.0: increased scale and scope after 10 years of protein dynamics modelling with python. *Bioinformatics*, 37(20):3657–3659.
